# Oxalate of Cerium as a Cough Remedy

**Published:** 1880-06

**Authors:** 


### OXALATE OF CERIUM AS A COUGH REMEDY.

Before the New York Therapeutical Society, Dr. Albert H. Smith reported cases illustrating the different degrees of success obtained in the use of oxalate of cerium in the treatment of cough. The conclusions reached were as follows:

1. Oxalate of cerium could be safely administered in doses of 10 grains three times a day for many days in succession.

2. The oniy unpleasant symptoms when so used was slight dryness of the mouth, that appeared after several days.

3. It was probably the most efficient when administered dry upon the tongue.

4. Its effects were not produced until two or three days after its use was begun and lasted for two or three days after the remedy was discontinued.

5. It was most efficacious in the treatment of chronic cough, and the initial dose should be 5 grains.

6. In the majority of cases it had not proved an efficient cough medicine for any considerable length of time, but could be regarded a valuable agent to be employed in alternation with other remedies.

7. It did not disturb the stomach; on the contrary, it relieved nausea and improved digestion.

8. Different preparations upon the market were not of equal value, and when success was not obtained by one, another should be substituted.—*Medical Record.*

